# Living with a Gastric Band: A Qualitative Study

**DOI:** 10.3390/healthcare2010047

**Published:** 2014-01-13

**Authors:** Michael Pfeil, Kenda Crozier, Amanda Pulford, Yasmin Ferguson, David Mahon, Michael Lewis

**Affiliations:** 1 School of Nursing Sciences, University of East Anglia, Norwich Research Park, Norwich, NR4 7TJ, UK; E-Mail: k.crozier@uea.ac.uk; 2 Spire Norwich Hospital, Old Watton Rd, Norwich, Norfolk, NR4 7TD, UK; E-Mail: amanda@pulford.fsnet.co.uk; 3 Department of Upper Gastrointestinal and Bariatric Surgery, Musgrove Park Hospital, Taunton and Somerset NHS Foundation Trust, Taunton, TA1 5DA, UK; E-Mails: yasmin.ferguson@tst.nhs.uk (Y.F.); david.mahon@tst.nhs.uk (D.M.); 4 Department of General Surgery, Norfolk and Norwich University Hospital NHS Foundation Trust, Colney, Norwich, NR4 7UY, UK; E-Mail: michael.lewis@nnuh.nhs.uk

**Keywords:** weightloss surgery, gastric banding, patient experience

## Abstract

Gastric banding is an established and effective form of weightloss surgery. Semi-structured interviews explored the experiences of gastric banding of twenty purposively recruited patients one year after surgery. Data was analysed using thematic analysis. Results: Three themes emerged. They included ‘Exercising choice’ (restriction by the band was counterbalanced by new food-related choices.); ‘Rediscovering life’ (improved health, physical ability and energy enabled the patients to re-discover life.) and ‘Goals achieved with no regrets’ (patients had nearly achieved their self-set goals.) Conclusion: Beyond achieving weight loss and improved health, the participants had improved quality of life as defined by patients. Knowledge about this active process informs the care of these patients.

## 1. Introduction

**Figure 1:**
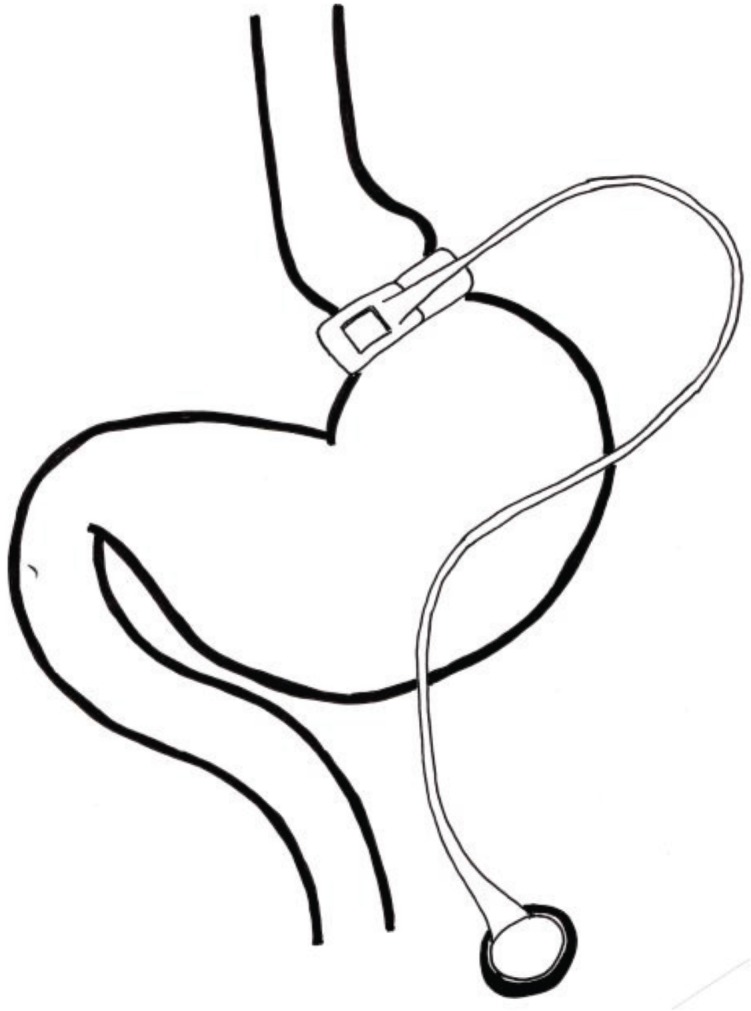
Laparoscopic Adjustable Gastric banding (LAGB).

Bariatric (weightloss) surgery is the most successful intervention in the long-term treatment of obesity and its comorbidities [1] with laparoscopic adjustable gastric banding (LAGB) being one of the most commonly used techniques [2]. The band is placed just below the gastro-oesophageal junction, creating a very small gastric pouch (Figure 1). The actual level of restriction by the band is determined by its ‘fills’, saline injections that tighten the band and so set the actual level of restriction. Gastric banding is a restrictive procedure, it reduces the amount of food that can be eaten without affecting the absorption of micronutrients and induces early and prolonged satiety through mediation by the intra-ganglionic laminar endings of the vagus nerve [3]. The possibility to remove the band also makes this a fully reversible procedure.

Gastric banding is comparatively safe [4], but poor adherence to dietary advice and follow-up can lead to complications, including oesophagitis, pouch dilation, and band erosion. Nevertheless, obese patients undergoing weightloss surgery live longer and with less comorbidities than those who do not [5]. They also experience significant psychological benefits [6].

Weightloss surgery (WLS) is not an easy option and for the patient it cannot mean opting out of the work of losing weight. Instead, having a gastric band is only the starting point in a chain of events that demand substantial lifestyle changes from these patients [7]. The patients’ input is therefore substantial and even crucial for the success of the intervention. The patients’ internal motivation to lose weight as well as their self-efficacy, autonomy, acceptance of responsibility in their lives, combined with their ability to handle life stress have been associated with success in WLS [8]. However, qualitative evidence concerning patient perceptions and experiences has only comparatively recently started to appear in the professional literature. The most notable issue arising from qualitative studies is that of control in relation to food and eating. This relates to patients hoping to achieve control [8], balancing on the edge of being in control and of losing it [9] as well as those reporting that the band makes them feel in control [10,11].

This study is part of the continuing effort to illuminate the patient experience of WLS.

## 2. Experimental

This qualitative study explored the patients’ experience of living with a LAGB as viewed by themselves 12 to 18 months after surgery.

The sample included two men and eighteen women aged 29 to 65 years, covering a wide range of educational backgrounds, including university graduates and others not holding any educational qualification. Recruitment took place at two sites, in Norfolk and Devon/Somerset. As during the recruiting period no patients of any other ethnic origin had bariatric surgery at the study sites, only participants with a white British background were recruited. Data were collected between August 2012 and February 2013 using semi-structured interviews lasting up to 60 min (interviewer: MP). The coverage of areas already identified in the existing literature as being of interest was ensured by using an interview schedule. Asking open-ended questions (See Table 1 for a sample question) allowed the participants to raise and pursue any issues of concern to them. The interviews were audio-recorded and transcribed verbatim.

**Table 1:** A sample question.

Question: Compared to one year ago: how do you feel now? Follow up prompts: What has changed? What made that difference? Why do you feel like that?

Thematic analysis was used to analyse the interview data. This is a commonly used approach in healthcare research, it is flexible and highly appropriate to find solutions for real world problems [12]. The open nature of the questions asked during the interviews allowed analysis to be purely inductive with all codes originating directly from data. The initial codes were refined, combined into themes and eventually united into three categories (see Table 2). Peer-debriefing tested the analytical results by exposing them to searching questions by experienced clinicians and researchers (K Crozier, A Pulford, Y Ferguson). Finally the finished categories were linked back to the original data to ensure that the analytical results were a true reflection of the data [13]. This is demonstrated by linking all direct quotations to the respective participant (*i.e.*, p10 = participant 10).

The transferability of qualitative research findings was increased by providing extensive data on the participant groups and study settings. It was enhanced further by collecting demographic information and quality of life data from all participants. The obesity-specific 31-item Impact of Weight on Quality of Life (IWQOL)-Lite questionnaire [14] is a psychometrically sound and clinically sensitive instrument with good content validity [15]. The numerical data were used to place the participants within the wider group of WLS patients. Statistical analysis was therefore limited to mean and standard deviation.

The research protocol was submitted to the South West Research Ethics Committee, Bristol (project identification code 11/H0203/6) and was approved on 1 March 2011).

**Table 2:** Emerging themes and categories.

**Theme 1: Having a band** Reduced food intake Tolerating different foods Meals in social settings Requiring support
**Theme 2: Exercising choice** Rejecting cheating Treating themselves Directing fills
**Theme 3: Re-discovering life** Improved health and abilities Exercise *versus* being active Body shape and looks Nice clothes Confidence
**Theme 4: Goals achieved with no regrets** Goals achieved or on the right track No regrets

## 3. Results and Discussion

### 3.1. Results

The results of the IWQOL-Lite questionnaire (see Table 3) showed comparability of the participants to the larger population of WLS patients. When compared to the pre-operative outcomes their results had improved throughout, with their post-operative results approaching those of the general population. Furthermore, a more confident body language was noticeable during many of the second interviews.

Having lost on average 33.2 Kg (range: 15.9–64.1 Kg) no participant regretted having had a gastric band inserted. Most had either achieved their goals or were close to doing so. Only two participants were ‘*a little bit disappointed*’ (p7), having shed 19 and 25 kg, they had hoped for faster weightloss. The interview results of this study can be grouped together into four overarching and inter-related categories.

#### 3.1.1. Having a Band

The gastric band was widely welcomed and accepted as ‘*a friendly thing*’ (p1) that ‘*has become part of my life*’ (p6) and ‘*is going to be with me forever*’ (p14). That the band enforced a restricted food intake was seen as its foremost benefit (*n* = 12). Overeating had become impossible (*n* = 10) because ‘*you haven’t got the capacity*’ (p23). Regurgitation, the result of eating too much, was disliked by all, but accepted as being ‘*just part of the process*’ (p6), an ‘*annoying*’ (p20) but ‘*reasonable price to pay*’ (p8). It served as a reminder of the limited stomach capacity and became less frequent as the participants adjusted to their new situation. The enforced control (p2) of their food intake was normally combined with a noticeable reduction in hunger (*n* = 10). ‘*Feeling full*’ (p13) helped not just to avoid eating, it combined with the perception that the lost weight ‘*will not come back*’ (p12) resulting in participants feeling ‘*liberated*’ by not having to ‘*think about food all the time*’ (p17).

**Table 3:** Results of the IWQOL-Lite [14].

	*Impact of Weight on Quality of Life Questionnaire-Lite* Mean and [SD]					
	overall	Physical function	Self-esteem	Sexual life	Public distress	work
**Scores, means and SDs according to participant group [14].**						
General community	91.8	90	87.5	95.1	96.5	95.4
	[12.0]	[14.9]	[19.4]	[13.0]	[10.9]	[11.5]
Severely obese	54.6	48.4	45.9	65.9	62	68.1
	[19.5]	[21.2]	[25.9]	[29.9]	[24.7]	[23.7]
Bariatric surgery	36.9	31.7	30.4	45.8	40.8	54.6
	[19.0]	[21.7]	[25.3]	[31.8]	[25.4]	[27.5]
**Scores produced for this study (*n* = 20): just before surgery**						
Mean	47.9	53.7	25.6	51.8	42	58.6
SD	22.1	19.6	25.4	34.8	28.7	20.6
**12 to 18 months after surgery**						
Mean	83.9	86.6	79.8	82.9	86.6	89.8
SD	16.75	16.59	16.48	18.01	20.48	13.68

There were, however, problems. Every participant could list a number of foods ‘*I can’t eat*’, mainly potatoes, rice, different meats, vegetables, salads and fruit. What exactly the problem foods were, varied from person to person. Furthermore, how much food the band allowed to pass varied from day to day. This individual tolerance of food and the ‘*fickleness*’ (p2) of the band were a further cause of regurgitation that presented the participants with a degree of unpredictability that could only be overcome by trial-and-error experiments.

Eventually the participants reached a point where they could ‘*judge portions*’ (p23) and pick food ‘*more consciously*’ (p9), but meals in social settings, for example visits to restaurants or family meals, could be problematic. Selecting food carefully (favouring soft food), small portion sizes (just having a starter) and paying particular attention to eating slowly were common coping strategies. ‘*Mishaps*’ (p14), *i.e.*, suddenly realising that they had to regurgitate and rushing to the toilet, did nevertheless happen and could be ‘*embarrassing*’ (p9).

Although the participants’ postoperative recovery was quick and uneventful, they required appropriate and specific information and advice to enable them to adjust to living with the band. As they found other health professionals (GPs, NHS help lines, Accident and Emergency departments) to have only limited knowledge, they viewed the bariatric nurses, who ‘*knew everything*’, to be their ‘*first port of call*’ for both routine and emergency advice (p10). Knowing that the nurse ‘*was at the end of a phone, if there were any problems or queries*’ (p18) and that ‘*you are never left on your own*’ (p17), was re-assuring, kept up motivation and was important for the overall success of the surgery. During the ‘*first few days*’ the contact between bariatric nurses and their patients was ‘*very close*’, with daily telephone calls. Overall the reliance on the bariatric nurse was greatest during the first six months after surgery and gradually diminished thereafter as the participants grew more knowledgeable about band-related issues and developed confidence in their own decision making.

#### 3.1.2. Exercising Choice

The band enforced substantial changes in the participants eating habits, but the knowledge of this restriction provided a feeling of ‘*security*’ as well as feeling ‘*in control*’ of their weight (p5). They were aware of ‘*ways around the band*’ (p3) for example by ‘*mushing everything up*’ (p3) or by ‘*grazing*’, issues that the participants had to deal with if they wanted to be successful. Resisting food required ‘*willpower*’ (p19) because ‘*the temptation is there* [and] *nothing stops you from wanting it*’ (p24). Many participants also knew people who did cheat, but rejected cheating for themselves to the point of feeling ’*quite angry*’ about those who did (p13). This does not mean that they always adhered 100% to the healthy eating advice and several admitted to this as a reason for their delayed or reduced weight loss (*n* = 3). However, the participants did not see the need to stop ‘*treating themselves*’ (p5).

*I do have desserts if I want to. If I go out for a meal and there’s an ice cream or whatever, and I’ve been good all week, then I treat myself*.

(p18)

Whether it was chocolate, chips, cakes, biscuits, ‘*a few drinks*’, sticky toffee pudding, cheese or ice cream, the participants enjoyed their ‘*treats*’ (p23). They did, however, consume ‘*much less of it*’ (p23) and it was this that distinguished a treat from cheating. This food was enjoyed without the participants losing control and without the food exerting power over the participants.

The actual level of restriction by the band was determined by its saline ‘*fills*’. Although advice regarding the fills was provided by the bariatric nurses, the participants had considerable influence over the fill levels administered. Many kept their band ‘*reasonably loose*’ to allow for the natural fluctuations in band tightness and steady weight loss (p2), but also to maintain their band-life balance.

*I could have another tightening, but I want a life as well*.

(p3)

Others urged healthcare staff to fill their band as soon and as tight as possible in order to achieve the biggest possible weightloss. This occurred especially when, following post-operative recovery weightloss was not instant.

*After five weeks, when you go back and put fluid in, I explained that I thought my portions were too big, because I’d lost only a few pounds. So she put some more fluid in and then that started to restrict how much I could eat*.

(p23)

To find the right balance was not easy and where the restriction became too severe (*n* = 3) the fill had to be reversed.

All in all the participants were aware that the band ‘*does not take choice totally away*’ (p14), they could still enjoy food and had considerable influence on how restrictive the band actually was. This ‘*working with the band*’ made participants feel ‘*in control of eating for the first time*’ (p5) because they could ‘*use the band as a tool*’ (p13) to ‘*live my life*’ (p2). 

#### 3.1.3. Re-Discovering Life

Control over their eating and the resulting weightloss resulted in positive changes, most noticeably in health as well as self-perception. Typically, type 2 diabetes mellitus, blood lipid levels, hypertension and arthritis in knees and ankles were resolved or improved.

*I was a diabetic and [now] all my bloods are normal. I take no diabetes medications whatsoever. I also had sleep apnoea and that has now gone*.

(p12)

These measurable improvements were a source of deep satisfaction and often relief, but the general gains in physical ability were seen as more important for everyday life. Only three participants exercised regularly in a gym and formal exercise regimes were frequently seen as ‘*really boring*’ (p14). Nevertheless, extended walks, whether with the ramblers or in the form of shopping trips were very popular and all reported often substantial improvements in physical fitness.

*I did have a running machine and an exercise cycle, but I used them less and less. Exercise now is based on the fact that I’m 67 years old, so it’s a good sturdy walk, gardening, pushing the mower around. My partner has an electric buggy, we switch it to high speed and I struggle to keep up. That’s exercise*.

(p17)

Increased overall activity levels were expressed as ‘*being on my feet*’ and ‘*on the go all day*’ (p8), daily ‘*brisk*’ walks’ (p1), always using the stairs rather than the lift (p12) and joining the Ramblers Association (p14). The increased activity resulted in being able to ‘*walk miles now*’ (p16), all ‘*around town*’ with ‘*no trouble*’, when before they ‘*had to keep stopping*’ (p18). The increased physical ability and the much wider range of activities that became possible as a result of being more fit were felt to be important signals of success.

*My most important moment was last year. I walked up to the local monument and as I was doing the last steps at the top I was still able to speak. I wouldn’t have been able to do that before. I don’t care how much I’ve lost, more important is that I can be active*.

(p14)

Similar importance was given to the change in body shape and looks caused by the reduction in weight. Statements asserting that they believed that they looked ‘*so much better*’ (p14), ‘*trimmer*’ (p17) and ‘*more attractive*’ (p5) were commonly made, at times with the admission that it was ‘*sheer vanity, really*’ (p9). Far from vanity, however, looking good normally meant feeling ‘*more normal*’ (p2) and ‘*like I fit in more*’ (p5). Not surprisingly the participants felt ‘*a big boost*’ (p18) and were ‘*quite happy with what’s in the mirror, really*’ (p23). The ‘*better looks*’ were enhanced by the much improved opportunities to buy new and nicer clothes, that made the participants ‘*feel good*’ instead of ‘frumpy’ (p18).

*It was lovely to go and buy new clothes. I can get into a size 16 now, it’s great. I go out now and I feel more confident in myself*.

(p24)

The importance of clothing was highlighted when four participants reported critical moments that they had lived through during the last year, to have been linked to clothes. This could either be ‘*going into a shop and buying smaller clothes*’ (p1) or ‘*going to the best friend’s wedding in a nice dress bought in a normal shop and showing [her] shape*’ (p3) as well as going down dress sizes and ‘*being able to buy the new clothes off the peg*’ (p4, p6). Although this choice of clothing could also be related to the ‘exercising control’ category above, the participants emphasized strongly the quality of life and self-perception aspects of this new part of their lives.

Once the weight loss occurred five participants found it difficult to recognise their own, changed body shape. Whether they still wanted to buy large clothes or only recognised their own weight loss when looking into a mirror or at a photograph, their body perception had not kept up with their new shape.

*I took a photo earlier and was quite surprised. In my own mind I still think I’m as big as I was before but to me the photo looked really good. I didn’t think I looked that nice, really*.

(p7)

For three participants the appearance of excess skin was a concern. While one participant tried to prevent it from happening by exercising and slower weight loss (p5), another did not have it removed because of her ‘*age*’ (p16). The third participant received further surgery to remove excess skin as ‘*completing the whole thing*’ (p8).

The most noticeable result of the combined changes was a marked increase in confidence among the participants. This could be expressed in the way they perceived themselves in the mirror, or how they felt in public.

*I feel generally more confident and I do tend to speak up more than I used to, because I am not so afraid of somebody looking at me. I feel more normal, essentially*.

(p2)

It is also most likely an expression of new confidence that four participants had entered into new relationships, with one getting married (p1), another having a new partner ‘*moving in*’ (p14) and a third planning to get married (p18).

*It was only once I had started to lose weight before the operation that I actually started looking and thinking that I perhaps could have another relationship*.

(p14)

#### 3.1.4. Goals Achieved with No Regrets

Although the participants spoke extensively about their new lives, they mentioned having achieved their self-set goals only briefly. However, they did so with great conviction. It is the strength of their feelings rather than the extent of the data that makes this category stand out and demonstrate its importance. Most participants (*n* = 17) were very happy with the weight loss they had achieved and declared that they had either reached their goal or were very close to it. They stated their individual main goal to be either their weight loss (*n* = 6), their improved health (*n* = 5) or the reduction in dress size (*n* = 6).

*My weight as a problem? Zero! There isn’t a problem now, my weight is not a problem at all*.

(p2)

Despite having shed between 19 and 25 kg, three participants had not yet achieved their self-set goals and so made more mixed statements. Two of them (p20, p22) still wished to lose another 18 to 24 Kg, while the third participant (p7) was already happy with her reduced waist line, but had hoped for a more pronounced weight loss. Having lost on average 33.2 Kg (range 15.9–64.1 Kg) no participant, including those whose weightloss turned out to be less than they had wished, regretted to have undergone gastric banding, but three participants wondered whether they should have had surgery earlier in their lives.

*Definitely no regrets, no concerns. If I could put the clock back I wonder how different my life would be if I’d had the band 10, 20 years ago*.

(p13)

### 3.2. Discussion

First and foremost the band controlled what and how much the participants could eat. The extent of the restriction fluctuated in its severity and was at times difficult to gauge due to its perceived ‘*fickleness*’ (p3), but was present around the clock. This control imposed on the patient and limited choice is well established in the literature [16]. Paradoxically having their food intake controlled by the band has been reported to result in a renewed sense of control by the participants [17]. The results of this study go further by explicating how the participants asserted a significant level of control over their band. This was expressed in several ways, for example the participants’ knowledge of ways to cheat the band. However, their rejection of cheating and submission to the impositions made by the band is evidenced by the weightloss achieved overall. Furthermore, some of the ‘*treats*’ that participants allowed themselves could theoretically be seen as cheating, but these transgressions remained minor and, without endangering the weightloss achieved, they provided a sense of increased quality of life as well as control. Lastly, the participants had considerable influence in deciding the severity of the band fills and therefore the amount of food the band would allow to pass. Although supported by guidance from the bariatric nurse, making this decision remained largely in the sphere of influence of the participants, who used this power to balance weight loss with their perceived quality of life.

The theme of control has been described as ‘*central*’ and ‘*permeating all areas*’ of the bariatric patient experience [14] (p273). However, in this study something else became obvious. The participants had accepted illness as well as treatment-driven boundaries into their lives [18], adjusted to them and thereby learned to live with them [19]. By doing so they felt that they had re-established control over their food intake [20] and therefore their obesity as well as its related problems. Not only accepting the restrictions imposed on them, but playing an active part in regulating their severity in order to achieve a band-life balance, resulted in the participants feeling empowered and actively changing their lives. This became most obvious in the second category, where the consequences of the resulting weightloss on the participants’ lives are illuminated.

WLS changed the participants’ life fundamentally. Their reduced weight resulted in the desired considerable improvements in health [19,20] and relieved their fears of future health deterioration. Similarly, the changed role of food in their lives [8] increased their appreciation of the food they now enjoyed and opened up more time for activities that made their lives more enjoyable.

The common lack of a formal exercise regime, a frequently asserted requirement for sustained weightloss, is likely to have reduced their weight loss [21]. However, the participants reported enjoying a dramatic and sustained increase in their general levels of activity, enabled by the weight loss achieved and the resulting increase in mobility and stamina. This improved quality of life was enhanced further by the changed body shape and improved body esteem [22], combined with a new ability to dress in nicer, better fitting clothes, all of which were highly appreciated changes. Everyday activities, including going shopping, playing with (grand) children, and going for long walks, that had been impossible were now re-discovered and greatly enjoyed. Most pronounced was the forming of new relationships by several participants, made possible by all these changes and the resulting self-esteem and confidence. Experiencing their new life also re-enforced their commitment to the band and assisted in maintaining the weightloss achieved [21].

Patient expectations towards gastric banding formed during their ‘journey’ to gastric banding have been described by us in an earlier study [23] (Table 4). The participants in this study successfully translated these expectations into post-operative achievements. They were able to control their food intake, and enjoyed improved health, increased mobility and stamina, as well as a better appearance. This enabled them to re-discover life and created a new sense of having overcome powerlessness and, most of all, they gained a sense of achievement.

**Table 4:** Patient expectations towards LAGB [23].

The gastric band is a tool, not a solution in itself. It helps to regain control and overcome powerlessness. The band will help me to achieve an increased ability to do things, an improved quality of life, being normal size, being able to wear nicer clothes that fit, having increased self-esteem, enjoying a healthier, longer life.

Apart from a lack of motivation, a lack of success following WLS has been attributed to an inappropriate diet and a lack of physical activity [24]. Both of these problems were avoided by the participants, although with some modification. They enjoyed ‘treats’, *i.e.*, small amounts of food types they were not supposed to eat, and most did not have a formal exercise regime, but reported being more physically active throughout their day. Both of these adjustments might have limited the amount of weight lost, but have been identified as contributing to a band-life balance that ultimately promoted success.

It is therefore not surprising that most participants in this study (21/24) had already achieved their gastric banding goals or were close to doing so. The three who had made progress but were still not close to their goal weight admitted that more of their own effort would have been needed to gain and maintain the desired weightloss. They attributed their failure to achieve their goals to not adhering to dietary and exercise advice. This confirms earlier findings that not all WLS patients can be expected to turn their awareness and knowledge into action [25].

#### Service Implications

The results of this study are of direct importance to health professionals and service providers. Most obviously the knowledge gained about the patient experience following gastric banding will enable nurses and surgeons to give better, more evidence-based advice. More importantly, the health professionals’ definition for success in weightloss surgery needs to be adjusted. Currently this is still measured in actual weight lost and cost saving to the health service [26]. For patients, however, these ‘outcome indicators’ make only limited sense. For those taking part in this study the focus of successful weightloss surgery centred on a feeling of empowerment and the re-discovering of life. In their view the reduction of the limitations imposed on them by being obese was much more important than just the number of kilogrammes lost.

This study did not aim at exploring the role of nurses in weightloss surgery. However, any service provider will find it necessary to ensure success for gastric banding patients by providing substantial nursing support, in terms of both quality and time available. Patients are expected to make decisions concerning their treatment. In order to exert control they need to have considerable knowledge and understanding relating to this type of surgery. Nurses need to be able to teach and advise, but also to allow their patients to arrive safely at decisions concerning their treatment. Beyond factual knowledge this demands excellent communication skills, enabling the nurse to instil this knowledge in the patient. Furthermore, the need to empower patients to make decisions themselves requires these nurses to be able to step back and relinquish control. When combined, these attributes call for a nurse of considerable experience and of nurse practitioner level [27]. During the early post-operative months patients will also require direct access to these suitably qualified nurses to manage any occurring crises, even if these do, in the eyes of outsiders, only constitute small concerns. Denying this type of access would result in patients seeking help from GPs and Accident and Emergency Departments, where suitable advice is uncertain and unnecessary hospital admissions might occur.

The need for a highly qualified practitioner to be available at all times does have considerable cost implications, but these could be offset by the overall savings to the health service inherent in successful weightloss surgery.

## 4. Conclusions

Gastric banding can change the quality of patients’ lives fundamentally. It works best for committed and knowledgeable patients. By placing the views and experiences of these patients centre-stage, this study provides some of the evidence required by healthcare professionals to provide well-informed support to bariatric patients.
